# A Novel *Smad7* Genetic Variant Mapping on the Genomic Region Targeted by Mongersen Is Associated with Crohn’s Disease

**DOI:** 10.3390/biomedicines8080234

**Published:** 2020-07-22

**Authors:** Davide Di Fusco, Irene Marafini, Carmine Stolfi, Edoardo Troncone, Sara Onali, Elisabetta Lolli, Flavio Caprioli, Stefano Mazza, Cascella Raffaella, Laura Manzo, Paola Borgiani, Paolo Giuffrida, Antonio Di Sabatino, Ivan Monteleone, Giovanni Monteleone

**Affiliations:** 1 Department of Systems Medicine, University of Rome “Tor Vergata”, 00133 Rome, Italy; di.fusco@med.uniroma2.it (D.D.F.); irene.marafini@gmail.com (I.M.); carmine.stolfi@gmail.com (C.S.); edotronk@hotmail.com (E.T.); elisabetta.lolli@uniroma2.it (E.L.); 2 Department of Biomedicine and Prevention, University of Rome “Tor Vergata”, 00133 Rome, Italy; sara.onali@uniroma2.it (S.O.); raffaellacascella@virgilio.it (C.R.); lauramanzo@hotmail.it (L.M.); borgiani@uniroma2.it (P.B.); ivan.monteleone@uniroma2.it (I.M.); 3 Gastroenterology and Endoscopy Unit, Fondazione IRCCS Ca’ Granda Ospedale Maggiore Policlinico, 20122 Milan, Italy; Flavio.caprioli@gmail.com (F.C.); stem311089@gmail.com (S.M.); 4 Department of Pathophysiology and Transplantation, Università degli Studi di Milano, 20122 Milan, Italy; 5 Molecular Genetics Laboratory UILDM, Santa Lucia Foundation, 00179 Rome, Italy; 6 Department of Internal Medicine, San Matteo Hospital Foundation, University of Pavia, 27100 Pavia, Italy; paolo.giuffrida01@gmail.com (P.G.); a.disabatino@smatteo.pv.it (A.D.S.)

**Keywords:** inflammatory bowel disease, Crohn’s disease, ulcerative colitis, Smad7, single nucleotide polymorphisms

## Abstract

Background: Down-regulation of Smad7 with a specific Smad7 antisense (AS) oligonucleotide-containing oral drug (Mongersen) was effective in pre-clinical studies and initial clinical trials in Crohn’s disease (CD) patients. A recent phase 3 trial was discontinued due to an apparent inefficacy of the drug, but factors contributing to the failure of this study remain unknown. Here, we analysed the frequency in CD of rs144204026 C/T single nucleotide polymorphism (SNP), which maps on the corresponding region targeted by the Smad7 AS contained in the Mongersen formulation and examined whether such a variant allele affects the ability of Smad7 AS to knockdown Smad7. Methods: rs144204026 SNP frequency was evaluated in two independent Italian cohorts of Crohn’s disease patients and normal controls. Genotyping was performed by allelic discrimination assay. *Smad7* expression was evaluated in *wild-type* or heterozygous PBMCs treated with Smad7 AS. Results: No TT genotype was seen in CD patients and controls. Heterozygous genotype was more frequent in CD patients of both cohort 1 (11/235, 4.68%) and cohort 2 (8/122, 6.56%) as compared to controls (6/363, 1.65%; *p* = 0.029 and *p* = 0.01 respectively). Overall, a statistically significant association was observed between the T variant allele and CD patients’ susceptibility (*p* = 0.008; OR = 3.28, 95%CI: 1.3–8.3). Smad7 AS down-regulated *Smad7* RNA independently of the presence of the variant allele. Conclusions: This is the first study to show an association between *Smad7* rs144204026 SNP and CD patients. Data indicate that such a variant does not negatively influence the in vitro inhibitory effect of Smad7 AS on Smad7.

## 1. Introduction

Crohn’s disease (CD) and ulcerative colitis (UC), the two major forms of inflammatory bowel diseases (IBD), are chronic immune-mediated disorders of the gastrointestinal tract the incidence of which is rising worldwide [1]. The aetiology of both CD and UC is unknown and no curative therapy still exists. However, in the last years several studies have contributed to delineate some of the major pathways of tissue damage in IBD, thereby facilitating the development of various therapeutic compounds which inhibit the mucosal inflammatory response in these disorders [1,2]. In this context, we have previously shown that the activity of transforming growth factor (TGF)-β1, one of the major counter-regulatory molecules, is severely impaired in the gut of IBD patients due to the elevated levels of the intracellular inhibitor Smad7 [3,4,5]. Smad7 represses TGF-beta signalling by stably binding to the cytoplasmic domain of activated type I TGF-β receptor and thereby competing with receptor-regulated Smads (i.e., Smad2/3). Additionally, Smad7 recruits the E3 ubiquitin ligases Smurf1 and Smurf2 to type I receptors to mediate ubiquitin-dependent degradation of the TGF-β receptor complexes [6,7]. Smad7-mediated inhibition of TGF-β1-driven Smad-dependent signalling is supposed to amplify inflammatory signals in the gut of patients with IBD [4]. Indeed, knockdown of Smad7 with a specific antisense oligonucleotide (AS) has been associated with restoration of TGF-β1/Smad signalling, reduced production of inflammatory cytokines by IBD mucosal cells and amelioration of gut inflammation in murine models of IBD-like colitis [4,8]. Consistently, a phase 1 study conducted showed that oral administration a Smad7 AS-containing oral drug, denominated Mongersen (previously termed GED-0301) in active CD patients was safe and associated with clinical benefit, and later on, two distinct phase 2 studies confirmed the safety of the drug and showed that Mongersen induced clinical and endoscopic improvement in steroid-dependent and/or resistant CD patients [9,10,11]. Despite these positive results, a recent phase 3 study was discontinued due to an interim analysis that documented the apparent clinical inefficacy of the drug [12]. The reasons for the discrepancy between the phase 3 study and the previous clinical trials remain to be ascertained even if differences in the patient criteria selection may have contributed.

Mongersen contains a phosphorothioate single-stranded AS matching the region 107–128 of the human *Smad7* complementary DNA sequence [3,4,5]. The ability of any AS to knockdown the specific RNA is strictly dependent on the precise binding of the oligonucleotide to its target [13]. Indeed, even single nucleotide mismatches between AS and its target can destabilize the hybrid, thus preventing the AS-driven target degradation [14].

A recent study has documented a *Smad7* single nucleotide polymorphism (SNP) rs144204026, which leads to a transition from Glycine to Arginine in position 39 into the MH1 domain of Smad7, in a father and son with extensive hamartomatous gastrointestinal polyposis and early-onset oesophageal cancer [15]. Notably, such a SNP maps on the corresponding genomic region targeted by Mongersen, raising the possibility that its presence can interfere with the ability of Smad7 AS to bind and suppress *Smad7* RNA expression.

This study was aimed at evaluating the frequency of the *Smad7* SNP rs144204026 C/T in CD and ascertaining whether such a SNP affects the ability of Smad7 AS to knockdown Smad7.

## 2. Experimental Section

### 2.1. Study Population

The study population included 697 IBD patients (357 CD and 340 UC) and 363 ethnically matched and healthy, unrelated blood donors, who were considered as controls (53% females; age 39 ± 14 years). Two IBD cohorts were considered: cohort 1 included 235 CD patients and 198 UC patients under regular follow-up at the Tor Vergata University Hospital (Rome, Italy); cohort 2 included 264 IBD patients (122 CD and 142 UC) followed at the San Matteo Hospital of Pavia (Pavia, Italy) and Ospedale Maggiore Policlinico (Milan, Italy). For each patient, several demographic and clinical variables were considered for the analysis (Table 1).

The research was approved by the Ethics Committee of “Tor Vergata” General Hospital of Rome (Ref. number: 174/15, approved on 19 September 2015) and was performed according to the Declaration of Helsinki. Informed consent was obtained from each individual.

### 2.2. DNA Extraction and Genotyping

DNA was extracted from whole blood of each patient and control by using a Qiagen blood DNA mini kit (Qiagen, Milan, Italy). Genotyping of *Smad7* SNP (rs144204026 C/T p.Gly39Arg) mapping on Chr.18:48.950.310 was performed by allelic discrimination assay by TaqMan technology (Thermo Fisher Scientific, Waltham, MA, USA) using ABI PRISM 7000. Known control genotypes were included in each run of the allelic discrimination assay. Heterozygous genotype of IBD patients and controls were confirmed by Sanger sequencing.

### 2.3. PBMCs Isolation and Culture

Peripheral blood mononuclear cells (PBMCs) were isolated from three *wild-type (wt)* (CC) three heterozygous (CT) individuals through density gradient centrifugation (Lympholyte-H; Cedarlane Labs, Burlington, Ontario, Canada,) according to the manufacturer’s procedures. PBMCs were checked for viability using 0.1% trypan blue and resuspended in X-VIVO 15 FBS-free medium (Lonza; Basel, Switzerland). To investigate whether the presence of the variant allele of rs144204026 associates with a reduced inhibitory effect of Mongersen on *Smad7* RNA expression, PBMCs were transfected with Smad7 AS (Mongersen) or sense oligonucleotide (both at 2 μg/mL final concentration) by Amaxa Nucleofector Technology (Lonza) according to manufacturer’s instructions and cultured for 18 h. TGF-β1 (2 ng/mL) was added to the cell cultures 30 min before the end of the treatment.

### 2.4. Assessment of Cell Death

To score cell death, PBMCs were either left untreated or treated with sense oligonucleotide or the Smad7 AS for 18 h. Cells were then collected, washed twice in PBS, stained with FITC-annexin V (AV, 1:100 final dilution, Immunotools, Friesoyte, Germany) according to the manufacturer’s instructions and incubated with 5 μg/mL of PI for 30 min at 4 °C. Fluorescence was measured using a Gallios (Beckman Coulter, Milan, Italy) flow cytometer. Viable cells were considered as AV−/PI− cells.

### 2.5. RNA Extraction and Real-Time PCR

Total RNA was isolated from PBMCs using PureLink Purification technology (Thermo Fisher Scientific). A constant amount of RNA was retrotranscribed into complementary DNA (cDNA). Reverse transcription was performed with Oligo (dT) primers and with SuperScript III Reverse Transcriptase (Thermo Fisher Scientific). Real-time PCR was performed using TaqMan gene assay (Thermo Fisher Scientific) using CFX-96 Real-Time PCR Detection System (Bio-Rad Laboratories, Milan, Italy).

### 2.6. Statistical Analysis

The Hardy–Weinberg equilibrium was verified by the Pearson Chi Square test. Differences in allelic and genotypic frequencies between IBD patients of cohort 1, 2 and controls were evaluated by the Pearson Chi Square test or by the Fisher Exact test, when required. In CD and UC patients, a genotype/phenotype correlation was investigated with the following clinical/demographic variables: CD location, CD behaviour, perianal disease, UC extent, gender proportion, age, smoking habit, and family history of IBD. Odds ratios (OR) with 95% Confidence intervals (CI) were also determined.

## 3. Results

### 3.1. Frequency of Variant Allele of rs144204026 in CD

To examine accurately the allele and genotype frequencies for rs144204026, we performed independent case/control association studies considering initially IBD patients referring at the IBD Center of Tor Vergata University in Rome (Cohort 1). For this purpose, DNA samples were collected from 235 CD patients and 198 UC patients and analysed for the rs144204026 SNP. Demographic and clinical characteristics of the IBD patients are shown in Table 1. No deviation from Hardy–Weinberg equilibrium for rs144204026 SNP was seen. No homozygous variant genotype (TT) was detected in IBD patients from Cohort 1. CT heterozygous genotype was more frequent in CD patients than in controls (11/235, 4.68% vs. 6/363, 1.65%; *p* = 0.029) while there was no difference between UC patients (7/198, 3.54%) and controls (*p* = 0.15) (Table 2). A statistically significant association between T variant allele and CD susceptibility was observed (*p* = 0.03; OR = 2.87, 95%CI 1.06–7.83), while no association was found for UC.

Next, we validated the above results using DNA samples taken from 122 CD patients and142 UC patients referring at two IBD centres in the Northern Italy (University of Pavia and University of Milan) (Cohort 2) (Table 2). Again, no TT homozygous genotype was detected in this cohort. The frequency of the CT heterozygous genotype was higher in CD patients (8/122, 6.56%), than in controls (*p* = 0.01) while there was no difference between UC patients (5/142, 3.52%) and controls (*p* = 0.19) (Table 2). T variant allele was associated with CD susceptibility (*p* = 0.03; OR = 4.06, 95%CI 1.39–11.8) but not with UC susceptibility. Overall, when the allelic frequencies from Cohort 1 and 2 were combined together, T variant allele was found to be associated only with CD and not with UC susceptibility (*p* = 0.008; OR = 3.28, 95%CI: 1.3–8.3 and *p* = 0.12; OR = 2.16, 95%CI: 0.8–5.8, respectively) (Table 3).

### 3.2. Relationship between Frequency of the T Variant Allele and the Demographic/Clinical Characteristic of CD Patients

Due to the low frequency of the T variant allele in the 2 cohorts of the study, we combined data from the two CD populations and ascertained whether there was a correlation of the CT heterozygous genotype with the demographic/clinical characteristic of the patients. No correlation between such a variant allele and the demographic and clinical variables of CD was identified, even if the carriers had more frequently lesions confined to the ileum (9/18, 50%) or ileum-colon (8/18, 44.4%) while only one carrier had colitis (Table 4).

### 3.3. Mongersen Down-Regulates Smad7 in PBMCs of Individuals Carrying T Variant Allele

**Figure 1:**
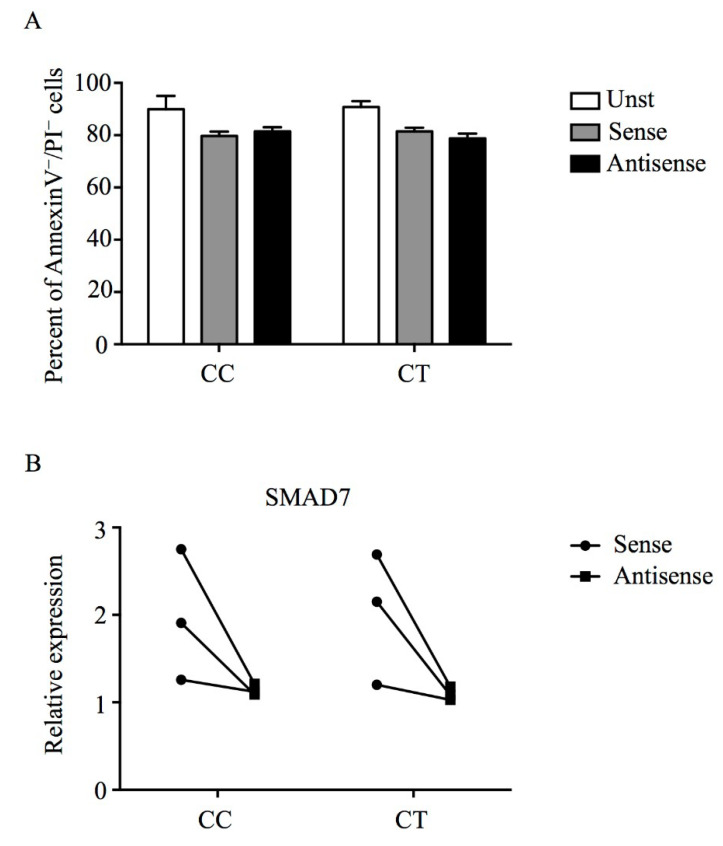
Smad7 antisense oligonucleotide does not induce cell death and inhibits *Smad7* expression independently of the presence of rs144204026 SNP. (**A**) PBMCs were isolated from 3 *wt* (CC) and 3 heterozygous (CT) healthy individuals and were either left untreated (Unst) or treated with Smad7 sense or antisense oligonucleotides by Amaxa Nucleofector Tecnology. Histograms show the percentage of viable cells considered as AnnexinV−/PI−cells assessed by flow cytometry after 18 h. Data are expressed as mean ± SD. (**B**) PBMCs from 3 *wt* (CC) and 3 heterozygous (CT) healthy individuals were transfected with Smad7 AS or sense oligonucleotide (both at final concentration 2 μg/mL) by Amaxa Nucleofector Tecnology and *Smad7* transcripts were evaluated by Real-time PCR. PBMCs were cultured for 18 h and rhTGF-β1 was added to the cell cultures 30 min before the end of the treatment. Levels are normalized to β-actin. Each point indicates the value of *Smad7* in a single cell sample.

To investigate whether rs144204026 genotypes affect the ability of Smad7 AS to down-regulate Smad7, PBMCs isolated from three *wt* (CC) and three heterozygous (CT) subjects were transfected with sense oligonucleotide or the Smad7 AS. For these studies we used the Smad7 AS contained in the Mongersen formulation. Transfection of PBMCs with Smad7 AS resulted in no induction of cell death (Figure 1A). As shown in Figure 1B, Smad7 AS down-regulated *Smad7* RNA transcripts independently of the presence of T variant allele.

## 4. Discussion

The data of this study demonstrate that the *Smad7* rs144204026 T variant allele was present in CD patients with a frequency significantly higher than in the control population and this finding was confirmed through the analysis of two distinct cohorts of patients. We found a statistically significant association between the rs144204026 T variant allele and CD susceptibility, thus confirming and expanding on data of previous studies showing a key role of Smad7 in amplifying inflammatory signals that contribute to the pathological process in this disorder [3,4,5]. In contrast, there was no association of the rs144204026 with UC. This is not surprising, as it has been reported that the 2 IBDs have a different genetic susceptibility and many SNPs have been associated with either CD or UC [16,17,18]. The presence of the *Smad7* rs144204026 T variant allele was associated with no demographic/clinical variable of the disease, even though the relatively low frequency of the variant could mask genotype/phenotype associations. In this context, however, it is noteworthy that virtually all the carriers of the variant allele had an involvement of the terminal ileum, raising the possibility that rs144204026 T variant allele could be an indicator of some subsets of patients with CD ileitis. It is not surprising that previous genome-wide association studies (GWAS) in IBD did not describe rs144204026 T variant allele. Indeed, GWA studies typically identify commonly occurring genetic variants, often located in non-coding regions, thus failing to pick-up those expressed at low frequency [19,20].

The relevance of rs144204026 C/T variant allele for CD pathogenesis remains to be ascertained. Previous immunohistochemical evaluation of esophageal tumour specimens demonstrated intact levels of Smad7 protein expression in the patients carrying on such a variant [15]. Therefore, we can speculate that the presence of the variant should not influence the content of Smad7 protein in inflamed gut of CD patients. However, this remains to be ascertained as we had no access to mucosal samples of patients carrying on rs144204026 variant. It is also conceivable that, independently of the protein content, the variant can affect either the cytosolic/nuclear distribution of Smad7 or Smad7 function. The crystal structure of Smad7 has not yet been determined and this makes it difficult to answer such a question.

Following the encouraging results of a placebo-controlled phase 2 trial of Mongersen showing a 55%–65% clinical remission rate at the highest doses of the drug [11], a phase 3 randomized double-blind, placebo-controlled, multicentre study was terminated early by the data monitoring committee as a consequence of a lack of efficacy [12]. Factors accounting for such a huge disparity between these trials remain unknown. It has been speculated that differences in inclusion criteria across the two trials could have contributed because, unlike the phase 3 study in which CD activity of the enrolled patients was documented by endoscopy and elevated levels of C-reactive protein and/or fecal calprotectin, the phase 2 study enrolment was based solely on the Clinical Disease Activity Index scores [21]. We think this is not sufficient to explain the differences between the 2 trials, as a recent phase 2 endoscopy study, in which patients with endoscopic lesions were enrolled, confirmed the benefit of Mongersen seen in the previous phase 2 study [9]. The phase 2 study enrolled patients from various centres in Italy and Germany, while the phase 3 study enrolled patients from across 34 countries in Asia, Europe, and North America. A possibility is that differences in genetic background of the patients might have contributed to the different results of these trials in line with the demonstration that some susceptibility genes are not equally represented in the IBD population across the world and response of CD patients to some drugs can be genetically influenced [22]. Since *Smad7* rs144204026 SNP maps on the genomic region targeted by Mongersen, we determined whether it interferes with the ability of the drug to knockdown *Smad7*, thus contributing to the explanation of the negative results of the phase 3 study with this drug. In PBMCs isolated from heterozygous individuals, a Smad7 AS similar to that contained in the Mongersen formulation inhibited *Smad7* expression at a similar extent of that seen in *Smad7 wt* PBMCs.

The findings of the present study show a novel association between *Smad7* rs144204026 SNP and CD susceptibility and demonstrate that such a variant allele does not alter the inhibitory effect of Smad7 AS.

## Figures and Tables

**Table 1:** Demographic and clinical characteristics of IBD patients.

Diagnosis	CD_1 N (%)	UC_1 N (%)	CD_2 N (%)	UC_2 N (%)	CD_Tot N (%)	UC_Tot N (%)
N	235	198	122	142	357	340
Gender Female	123 (52.3)	86 (43.4)	49 (40.2)	49 (34.5)	172 (48.2)	135 (39.7)
Mean Age	48	55	51	51	50	52
Smoking habit						
Current	64 (27.2)	24 (12.1)	18 (14.8)	2 (1.4)	82 (23)	26 (7.6)
Former	47 (20)	44 (22.2)	13 (10.7)	15 (10.6)	60 (16.8)	59 (17.4)
Family history of IBD	48 (20.4)	27 (13.6)	7 (5.7)	3 (2.1)	55 (15.4)	30 (8.8)
CD location *						
Ileum	136 (57.9)	-	39 (32)	-	175 (49)	-
Colon	15 (6.4)	-	19 (15.6)	-	34 (9.5)	-
Ileo-colon	64 (27.2)	-	64 (52.5)	-	128 (35.9)	-
Isolated upper disease	5 (2.1)		-		5 (1.4)	-
CD behaviour **						
Non-structuring/ Non-penetrating	63 (26.8)	-	60 (49.2)	-	123 (34.5)	-
Stricturing	100 (42.6)	-	26 (21.3)	-	126 (35.3)	-
Penetrating	57 (24.3)	-	23 (18.9)	-	80 (22.4)	-
Perianal disease	73 (31.1)	-	22 (18.0)	-	95 (26.6)	-
Colitis extent ***						
Distal	-	95 (47.9)	-	49 (34.5)	-	144 (42.3)
Left sided	-	28 (14.1)	-	38 (26.7)	-	66 (19.4)
Extensive	-	60 (30.3)	-	55 (38.7)	-	115 (33.8)

CD_1 and UC_1: IBD patients of Cohort1 (Rome, Italy). CD_2 and UC_2: IBD patients of Cohort2 (Pavia and Milan, Italy). * CD: 15/357 patients without a detailed history including CD localization. ** CD: 28/357 patients without a detailed history including CD behaviour. *** UC: 15/340 patients without a detailed history including UC extent.

**Table 2:** Genotypes association analysis.

	Controls N = 363 (%)	CD_1 N = 235 (%)	UC_1 N = 198 (%)	CD_2 N = 122 (%)	UC_2 N = 142 (%)	CD_Tot N = 357 (%)	UC_Tot N = 340 (%)
CC	357 (98.3)	224 (95.3)	191 (96.5)	114 (93.4)	137 (96.5)	338 (94.7)	328 (96.5)
CT	6 (1.7)	11 (4.7)	7 (3.5)	8 (6.6)	5 (3.5)	19 (5.3)	12 (3.5)
TT	0	0	0	0	0	0	0
		*p* = 0.029	*p* = 0.15	*p* = 0.01	*p* = 0.19	*p* = 0.007	*p* = 0.11

CD_1 and UC_1: IBD patients of Cohort1 (Rome, Italy). CD_2 and UC_2: IBD patients of Cohort2 (Pavia and Milan, Italy).

**Table 3:** Alleles association analysis.

	Controls N = 726 (%)	CD_Tot N = 714 (%)	UC_Tot N = 680 (%)
C	720 (99.2)	695 (97.3)	668 (98.2)
T	6 (0.8)	19 (2.7)	12 (1.7)
		*p* = 0.008 OR = 3.28 (1.3–8.3)	*p* = 0.12 OR = 2.16 (0.8–5.8)

**Table 4:** Genotype/phenotype association study in CD patients.

Diagnosis	CC N = 338 (%)	CT N = 19 (%)	*p* Value
Gender Female	163 (48.2)	10 (55.5)	*p* = 0.54
Mean Age	50	50	
Smoking habit			
Current	78 (23.1)	4 (22.2)	*p* = 0.93
Former	57 (16.9)	3 (16.7)	*p* = 0.98
Family history of IBD	52 (15.4)	3 (16.7)	
CD location *			
Ileum	166 (49)	9 (50)	*p* = 0.94
Colon	33 (9.5)	1 (5.6)	*p* = 0.55
Ileo-colon	120 (35.9)	8 (44.4)	*p* = 0.44
Isolated upper disease	5 (1.4)	-	
CD behaviour **			
Non structuring/ Non penetrating	116 (34.5)	7 (38.9)	*p* = 0.69
Stricturing	121 (35.3)	5 (27.8)	*p* = 0.48
Penetrating	74 (22.4)	6 (33.3)	*p* = 0.25
Perianal disease	92 (27.2)	3 (16.7)	*p* = 0.32

* CD: 15/357 patients without a detailed history including CD localization. ** CD: 28/357 patients without a detailed history including CD behaviour. Clinical data of 1/19 CT patient are missing.
